# Another string to the polo bow: a new mitotic role of PLK1 in centromere protection

**DOI:** 10.1080/23723556.2019.1658515

**Published:** 2019-09-17

**Authors:** Tomisin Olukoga, María Fernández-Casañas, Kok-Lung Chan

**Affiliations:** Genome Damage and Stability Centre, University of Sussex, Brighton, UK

**Keywords:** PLK1, chromosome biorientation, centromere dislocation, ultra-fine DNA bridges, BLM helicase, PICH translocase

## Abstract

Polo-like kinase 1 (PLK1) plays a fundamental role in the spatiotemporal control of mitosis. Cells lacking PLK1 activity exhibit characteristic chromosome misalignment due to defects in microtubule-kinetochore organization and attachment. In our recently published paper, we uncover a new role for PLK1 in the preservation and maintenance of centromere integrity.

Faithful segregation of chromosomes during mitosis is required by all cellular organisms to maintain their genomic balance. Errors in this process can have severe consequences leading to deleterious aneuploidies and chromosomal instability;^1^ features often observed in cancerous cells. In order to segregate chromosomes equally, mitotic cells must achieve chromosome biorientation; a process in which chromosomes are correctly attached to spindle microtubules emanating from opposite centrosomes at sister kinetochores, which promote proper metaphase alignment. As mitosis progresses, the metaphase to anaphase transition is tightly controlled by the spindle assembly checkpoint (SAC), which monitors any mis- and unattached chromosomes, inhibiting premature activation of the anaphase promoting complex/cyclosome (APC/C) and blocking anaphase onset. Once biorientation is achieved on every single chromosome, the SAC is silenced, leading to APC/C activation and the cleavage of cohesin. As a result, synchronous movement of the disjoined sister chromatids to the opposite poles occurs and the goal of equal chromosome segregation is reached. Centromeres play an indispensable role during both chromosome biorientation and segregation. They provide the foundation for assembly of the kinetochore and recruitment of signaling components that ensure proper kinetochore-microtubule (KT-MT) attachments.^2^ Moreover, they also serve as the major site for the cohesin complex to hold sister chromatids together.^3^

The serine/threonine protein kinase Polo has been reported to be essential for chromosome biorientation.^4^ Polo kinase was initially identified by analysis of Drosophila melanogaster mutants that exhibited mitotic defects.^5^ Since its discovery over 30 years ago, Polo and other members of the Polo-like kinase (PLK) family have been shown to be master regulators in multiple important aspects of mitosis. PLKs are evolutionarily conserved among different species; budding yeast and fission yeast have cell division cycle 5 (Cdc5) and Plo1 respectively, Drosophila has Polo and Polo-like kinase 4 (PLK4), whilst five PLKs have been discovered in humans. Of the five PLKs in humans, Polo-like kinase 1 (PLK1) is the most well characterized.^6^ It is now clear that the correct localization of PLK1 to diverse mitotic structures including centrosomes (for centrosome maturation), kinetochores (for KT-MT attachment), the central spindle and midbody (for spindle elongation and cytokinesis) is important for its functions. In early mitosis, PLK1 localizes to kinetochores, and its high activity there facilitates stable kinetochore-microtubule attachments.^6^ Loss of function of PLK1 leads to mitotic cell arrest in a prometaphase-like state with distinct monopolar spindles and a chromosome misalignment pattern (so-called ‘polo’ misalignment). This phenotype is generally attributed to a failure in the maintenance of stable KT-MT attachment.

Although the mechanism by which PLK1 promotes chromosome biorientation is still yet to be fully elucidated, in our recent study, we uncover an alternative mechanism explaining chromosome misalignment in PLK1-inactive cells^7^ (Figure 1). We demonstrated that in the absence of PLK1, and under bipolar spindle pulling, the DNA translocase Plk1-interacting checkpoint helicase (PICH) and Bloom’s syndrome helicase (BLM), aberrantly target and unwind centromeric chromatin into a thread-like DNA structure reminiscent of anaphase ultra-fine DNA bridges (UFBs). Consequently, centromere architecture is destroyed, leading to separation of the short and long arms of chromosomes and a characteristic metaphase misalignment pattern (named ‘figure 8-like’ pattern). This finding, in addition to shedding light on an alternative mechanism of chromosome misalignment, also reveals a previously undescribed role of PLK1 to counteract a centromere-specific breakage pathway. One that is distinct from other possible causes of centromere breakage (reviewed in^8^). Since polo kinases are evolutionarily conserved, it would be interesting to see if this new mitotic role is also conserved. However, PICH orthologues have not been identified in typical invertebrate model organisms (yeasts, Drosophila and Caenorhabditis), suggesting that this centromere-protective function may be species-specific, but further investigation is required.

**Figure 1.**
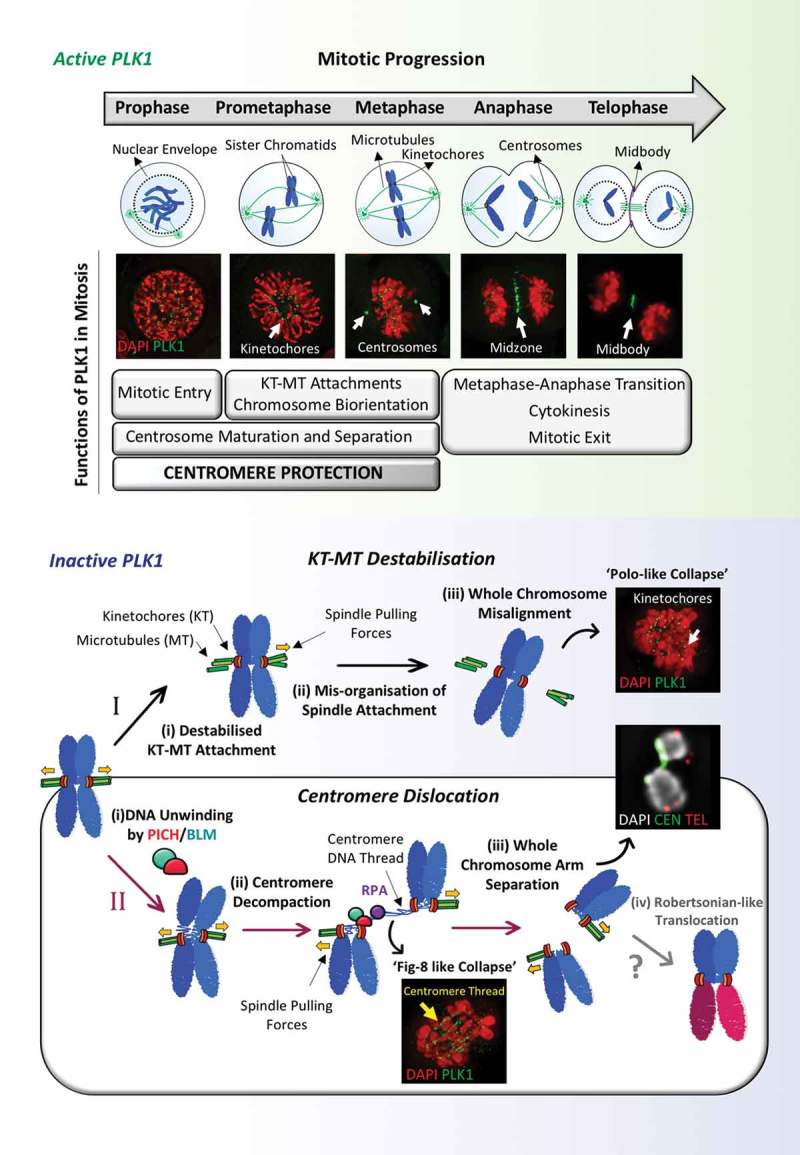
A new mitotic function of Polo-like kinase 1(PLK1). In addition to the existing roles of PLK1 in mitosis, we demonstrate that it confers an unexpected function to protect centromere integrity for chromosome alignment. (I) In the absence of PLK1 activity, kinetochore-microtubule (KT-MT) attachments are destabilized, leading to whole chromosome misalignment. This manifests as a ‘polo-like’ misalignment pattern. (II) If the polar KT-MT connection is not fully compromised, centromeres are aberrantly targeted by Bloom’s syndrome helicase (BLM) in a PLK1-interacting checkpoint helicase (PICH)-dependent manner. This leads to unlawful and excessive formation of ssDNA coated by replication protein A (RPA) which alters centromere configuration. Forces exerted by the bipolar spindle attachment pull out the centromere chromatin, which might trigger further DNA unwinding by the PICH/BLM complex. As a consequence, the centromere axis is decompacted, leading to the formation of centromere DNA threads and whole-chromosome arm separation. Cells therefore fail to maintain chromosome biorientation and result in metaphase collapse. This manifests as a ‘fig 8-like’ misalignment pattern. The centromere-specific chromosomal rupture could potentially lead to whole-arm (Robertsonian-like) translocations in segregating cells.

Numerical and structural alterations are known to be a common feature of most, if not all, tumor cells. It is believed that they contribute to the early steps of tumourigenesis and are implicated in cancer heterogeneity. Interestingly, many types of tumor that show a high degree of chromosomal instability (CIN) also exhibit chromosomal rearrangements at centromeres (reviewed in^8^). We show that PLK1 plays a crucial role in preventing devastating centromere damage and whole chromosome arm splitting. It is conceivable then, that when PLK1 activity is blocked (even transiently), the resulting ‘centromere-damaged’ chromosome arms may act as an ideal precursor for Robertsonian (-like) translocations – a form of whole-arm rearrangement. Given the important roles of PLK1 for chromosome segregation and centromere maintenance, it is not surprising that it is highly upregulated in human tumors^9^ and is considered as a prime therapeutic target.^10^ Various potent small molecule inhibitors have been developed against PLK1 and have shown promise in clinical trials.^10^ However, in light of our recent finding of PLK1 in centromere integrity maintenance, the use of PLK1 inhibitors in cancer therapies may need to be re-visited. It could potentially act as a double-edged sword. On one hand, in addition to the block of mitotic progression, the centromere-specific damage induced by PLK1 inhibition might trigger further catastrophic mitotic responses in tumor cells, thus enhancing the efficacy of PLK1 inhibitor treatments. On the other hand, if whole-arm translocations can be initiated through the centromere-breakage pathway, then long-term use of PLK1 inhibitors may promote unwanted chromosome rearrangements in normal cells that may lead to transformation. Nevertheless, as a key regulator of mitosis, a greater understanding of the exact mechanisms by which PLK1 executes its multifaceted functions might aid the development of new therapeutic approaches targeting the protein.

In conclusion, it is evident that preservation of centromeres is required to ensure faithful chromosome segregation and the maintenance of their integrity is of paramount importance to our cells. We highlight a new centromere protective role for PLK1, and our study provides a platform for further exploration of the pathways of centromere maintenance in mitosis and offers insights into its importance on genome stability.
